# Non-motor Characterization of the Basal Ganglia: Evidence From Human and Non-human Primate Electrophysiology

**DOI:** 10.3389/fnins.2018.00385

**Published:** 2018-07-05

**Authors:** Robert S. Eisinger, Morgan E. Urdaneta, Kelly D. Foote, Michael S. Okun, Aysegul Gunduz

**Affiliations:** ^1^Department of Neuroscience, University of Florida, Gainesville, FL, United States; ^2^Department of Neurosurgery, Center for Movement Disorders and Neurorestoration, University of Florida, Gainesville, FL, United States; ^3^Department of Neurology, Center for Movement Disorders and Neurorestoration, University of Florida, Gainesville, FL, United States; ^4^Department of Biomedical Engineering, University of Florida, Gainesville, FL, United States

**Keywords:** basal ganglia, electrophysiology, non-motor, deep brain stimulation, subthalamic nucleus

## Abstract

Although the basal ganglia have been implicated in a growing list of human behaviors, they include some of the least understood nuclei in the brain. For several decades studies have employed numerous methodologies to uncover evidence pointing to the basal ganglia as a hub of both motor and non-motor function. Recently, new electrophysiological characterization of the basal ganglia in humans has become possible through direct access to these deep structures as part of routine neurosurgery. Electrophysiological approaches for identifying non-motor function have the potential to unlock a deeper understanding of pathways that may inform clinical interventions and particularly neuromodulation. Various electrophysiological modalities can also be combined to reveal functional connections between the basal ganglia and traditional structures throughout the neocortex that have been linked to non-motor behavior. Several reviews have previously summarized evidence for non-motor function in the basal ganglia stemming from behavioral, clinical, computational, imaging, and non-primate animal studies; in this review, instead we turn to electrophysiological studies of non-human primates and humans. We begin by introducing common electrophysiological methodologies for basal ganglia investigation, and then we discuss studies across numerous non-motor domains–emotion, response inhibition, conflict, decision-making, error-detection and surprise, reward processing, language, and time processing. We discuss the limitations of current approaches and highlight the current state of the information.

## Introduction

The putative role of the basal ganglia as a predominantly motor structure in the brain is being increasingly challenged. Several lines of evidence implicate these nuclei in a comprehensive and expanding list of non-motor areas–emotion, language, decision-making, learning, memory, and more (Saint-Cyr, 2003; Weintraub and Zaghloul, 2013). Nowadays, mesocorticolimbic structures such as the ventral striatum are generally accepted non-motor foci in the brain, particularly within the realm of reward systems. However, a potential non-motor role for other key basal ganglia structures and the nigrostriatal pathway have only recently emerged. A new body of evidence demonstrates both motor and non-motor activity in what was previously considered purely motor territory of the basal ganglia nuclei. While multiple prior reports have summarized such evidence from non-primate animal studies (Baunez and Lardeux, 2011; Weintraub and Zaghloul, 2013; Rossi et al., 2015), human behavioral or imaging studies (Weintraub and Zaghloul, 2013; Voon et al., 2017), and computational modeling or simulation studies (Wiecki and Frank, 2013; Mandali et al., 2016), here we turn our attention to non-human primate and human electrophysiology recordings.

**Figure 1:**
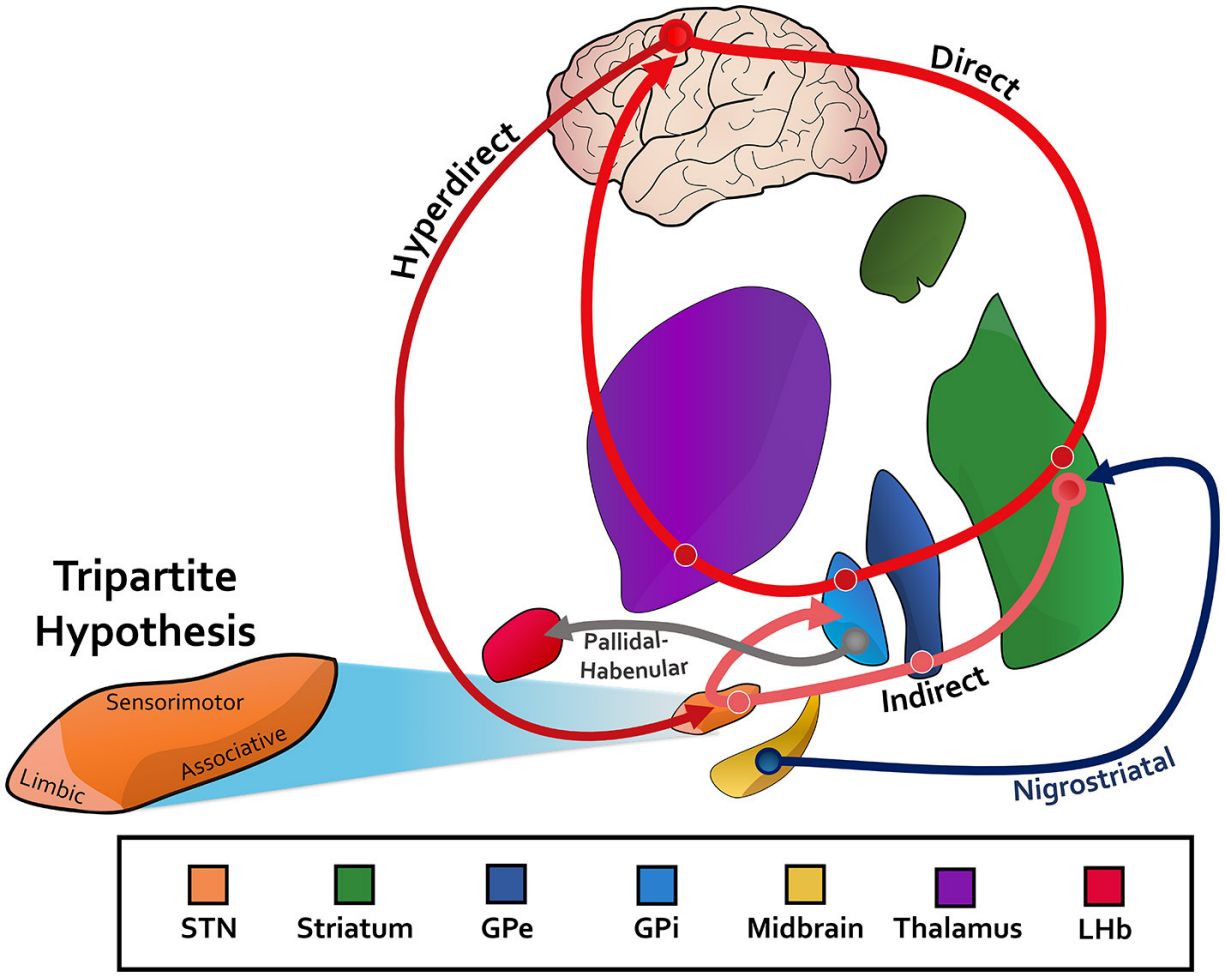
STN, subthalamic nucleus; GPe, globus pallidus externus; GPi, globus pallidus internus; LHb, lateral habenula. The canonincal basical ganglia model, with an associated direct, indirect, and hyperdirect pathway. Pathways between the midbrain and striatum (nigrostriatal) for dopaminergic innervation are depicted. The reward-relevant pathway between the pallidum and LHb is also included. The tripartite hypothesis is shown for the subthalamic nucleus as an example, in which the ventromedial aspect is for limbic function, the dorsolateral aspect for motor function, and the ventrolateral aspect for associative function. See Text for more information.

The basal ganglia communicate with higher-order cortical regions through a direct, hyperdirect, and indirect pathway (Figure 1). The direct pathway releases globus pallidus internus (GPi) mediated inhibition of the thalamus while the hyperdirect cortical input to the subthalamic nucleus (STN), and the indirect pathway via the globus pallidus externus (GPe) and STN, suppress thalamic activity through GPi activation (Nambu et al., 2002; Saint-Cyr, 2003; Aron et al., 2016). With respect to the motor cortex, these competing pathways comprise the balancing act critical for normal movement. In addition to motor function however, the basal ganglia nuclei and associated pathways are hypothesized to support distinct but parallel associative and limbic loops as well, each encompassing their respective cortical substrate (Figure 1; Temel et al., 2005; Okun, 2013). While early studies established this so-called tripartite division of the basal ganglia nuclei (Figure 1), more recent work has challenged the notion of distinct anatomic boundaries between motor and non-motor regions. Nonetheless, it is through these additional pathways that measurable non-motor processes exert their effect on human behavior.

Motivated largely by animal work, researchers have investigated non-motor electrophysiology of the basal ganglia in non-human primates for more than four decades (Travis et al., 1968; Hollerman et al., 1998). Human recordings, on the other hand, are relatively few in number due mainly to methodological difficulties. Deep brain stimulation (DBS), an FDA approved treatment for several neurological and psychiatric disorders, offers unprecedented opportunity to study *in vivo* human function of the basal ganglia. Implanted electrodes that deliver current to the brain may also be used for signal acquisition. The surgical procedure thus enables access to electrophysiological recordings of the deep brain in an awake individual, opening doors for intraoperative research endeavors of basal ganglia anatomy and physiology. Some institutions also conduct studies with DBS patients postoperatively, when wires are externalized and time constraints are less prohibiting.

Today the majority of DBS cases worldwide are performed on Parkinson's disease (PD) patients for which the most common brain targets include the STN and GPi (Okun, 2013). Hence, recent human electrophysiological studies have primarily relied on a PD DBS model to investigate both motor and non-motor processing in the basal ganglia, usually within the STN. Identifying and characterizing the underlying physiologic processing of non-motor function is a highly important area of research. Beyond basic neuroscience investigation, this line of work has important clinical implications for a wide range of neurological and psychiatric disorders. For instance, it may elucidate the observed improvements or deteriorations in non-motor symptoms seen after DBS for a broad range of FDA-approved and experimental uses (Parsons et al., 2006), and offer insights for more selective neuromodulation therapies (Urdaneta et al., 2017).

In this review, we focus on electrophysiological evidence for non-motor function in human and non-human primate basal ganglia, primarily of the STN. We briefly introduce the commonly used electrophysiological technologies in the first section of this paper and then refer to each technology throughout the text. Separate non-motor domains are addressed in dedicated sections. For each domain, we discuss the current state of knowledge and point out gaps or areas that future research could address. We conclude by discussing the limitations of current approaches and by highlighting the wealth of information that these studies have provided.

## Electrophysiological methodologies

**Figure 2:**
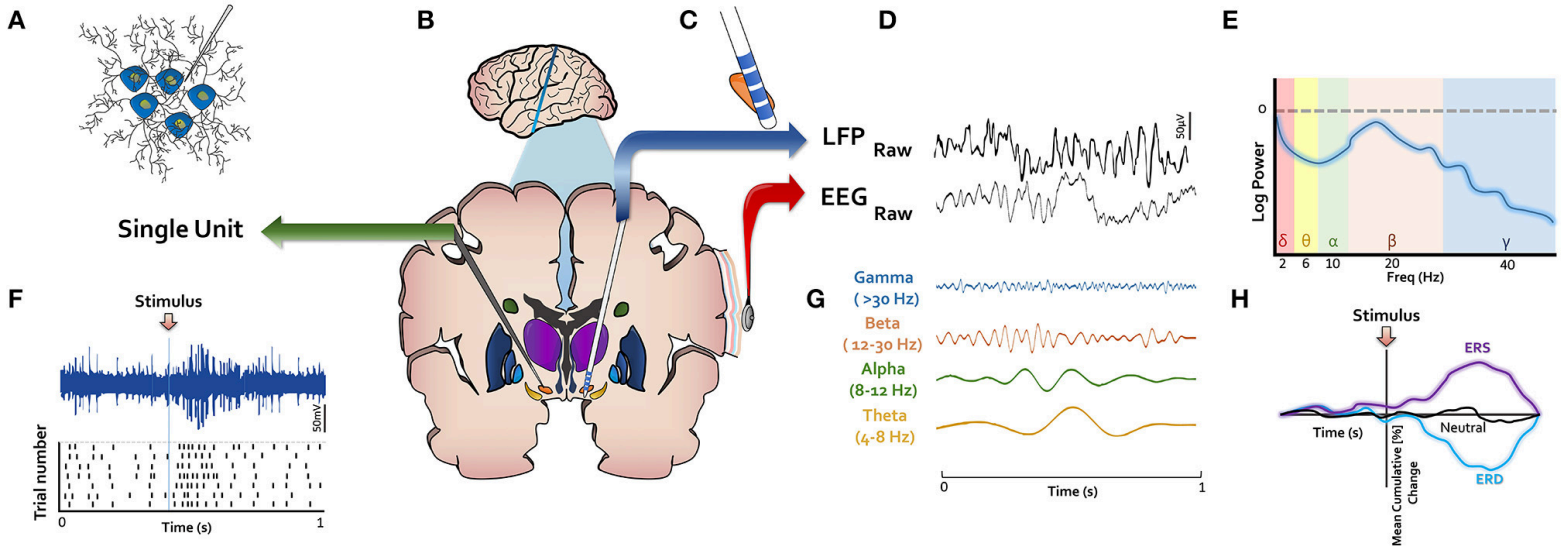
LFP, local field potential; ERS, event related synchronization; ERD, event related desynchronization. Methodological techniques for electrophysiological investigation of basal ganglia depicted in **(B)**. **(A)** Microelectrodes record single- or multi-unit activity and capture their action potentials as shown in **(F)**. **(C)** Macroelectrodes are implanted permanently in DBS surgery in basal ganglia nuclei. **(D)** Macroelectrodes yield LFPs, which have similar spectral content as EEG **(E,G)**. **(H)** Event-related changes in signals (ERPs) or oscillatory power (ERS or ERD) are of interest in studying response to stimuli.

Electrophysiological studies of the basal ganglia primarily analyze brain signal from either a microelectrode or a macroelectrode array (Figure 2). In the case of DBS, the macroelectrode remains permanently implanted within the targeted structure, whereas temporary microelectrodes are inserted at some centers for functional targeting as a preliminary step prior to final macroelectrode placement. Microelectrode recordings generally represent the activity of one (i.e., single unit) or a few neurons (i.e., multi-unit) in the surrounding region (Figure 2A). Macroelectrode recordings on the other hand capture local field potentials (LFPs), which are thought to represent the weighted average of many neurons located within a micron-scale region (Figure 2C; Buzsáki et al., 2012). Electrocorticographic (ECoG) signals are similarly field potentials acquired via subdural electrode arrays. Moving more superficially, non-invasive technologies such as electroencephalography (EEG) (Figure 2D) and magnetoencephalography (MEG) also represent the weighted activity of larger populations of neurons. While these recording techniques offer high temporal precision, they can differ substantially in their spatial resolution. To overcome this limitation, a few recent studies have employed several of these technologies simultaneously (Litvak et al., 2011; Zavala et al., 2014).

The most common methodologies used to analyze or interpret the signals from these recording devices are depicted in Figures 2E–H. Action potentials from single unit data are frequently represented in raster plots centered at an event of interest (e.g., when a stimulus is presented to a participant) (Figure 2F), with each row corresponding to a single trial. In this approach, the difference in neuronal firing pattern before and after an event of interest can be illustrated. Significant changes in firing rate can indicate that the recorded neuron's activity relates to the event of interest. Given the alikeness of the spectral content of LFP and EEG (Figure 2D), analytic approaches applied to their waveforms are generally similar. The raw data is routinely decomposed into oscillatory frequencies (Figure 2G), which are commonly categorized into different frequency bands. Although there are minor differences in band definitions, commonly used cutoffs include: delta (1–4 Hz), theta (4–8 Hz), alpha (8–12 Hz), beta (12–30 Hz), and gamma (>30 Hz). For a given segment of signal, the power (i.e., energy) across many different frequencies can be visualized or quantified using a power spectral density curve (Figure 2E). Some studies also analyze signals from multiple sources (e.g., LFP and EEG) to understand coherence between distant structures in the brain. The power within a specific frequency range over time may also be computed and visualized on a time-axis (Figure 2H). If this axis is centered on an event of interest, an event-related desynchronization (ERD), or synchronization (ERS) may be seen, which corresponds to a decrease or increase in power, respectively. More simply, if the raw signal is plotted on a time axis around an event of interest, an event related potential (ERP) may be seen. A significant power offset may be referred to by the approximate time point at which it occurs (e.g., P300 or N300 for a positive or negative deflection at 300 ms, respectively) or to the relative peak within an ERP (e.g., P3 for the third positive deflection). We refer to these various analytic modalities throughout this review.

## General non-motor processing

One of the early human electrophysiological studies supporting a potential role for the basal ganglia in cognitive function is from Rektor and colleagues (Rektor et al., 2005). The researchers recorded ERPs of various auditory and visual stimuli from macroelectrodes distributed throughout the caudate, putamen, or pallidum in 9 epilepsy patients. Participants were asked to engage in different cognitive tasks, such as recognizing or counting target tones. In all recorded locations, a P3-like wave was observed. Critically, this observation held when participants were engaged in tasks without any movement. To the authors' surprise, unlike the tripartite hypothesis, anatomically focal areas showing non-motor responses within these structures were not identified. Although the precise behavioral significance of a P3-like wave is not fully known, these results hinted that the basal ganglia may have an electrophysiologically measurable contribution to non-motor processing. When the paradigm was modified to include an increased cognitive load, similar STN recordings of P3-like potentials showed increased amplitude and latency (Baláz et al., 2008; Rektor et al., 2009). These results suggested non-specific cognitive processing of information within the STN. Future studies would soon elucidate more specific non-motor basal ganglia functions, but this work provided clear evidence to catapult further investigations.

The overwhelming majority of non-motor electrophysiological studies of the basal ganglia utilize variants of common computer tasks typically used for behavioral studies. Such tasks attempt to isolate brain signals during specific motor and non-motor events. But studies of the so-called default mode network, when participants are at rest and disengaged from purposeful behavior, also provide important evidence for non-motor function in the basal ganglia. In one such study, STN LFP and cortical MEG signals were simultaneously acquired from 13 PD patients at rest a few days after DBS surgery (Litvak et al., 2011). The temporoparietal cortex and STN were coherent within an alpha frequency (7–13 Hz) range, and the frontal cortex and STN were coherent within a high beta frequency (15–35 Hz) range. In light of the cortical areas communicating with the STN, these distinct networks likely represent substrates of attentional and executive processes, respectively. Although these basal ganglia-cortical projections have been established with numerous methodologies, it remains to be determined whether these specific communication frequencies are completely physiological or influenced by disease pathology. Repeat studies are needed across a range of patient populations.

## Emotion

Motivated by reports of PD DBS side effects such as depression, anxiety, emotional dysfunction, mania, aggression, and apathy (Kim et al., 2015), Kühn and colleagues were first to directly characterize the electrophysiological activity of the STN during emotional processing (Kühn et al., 2005a). Emotion content customarily consists of two dissociable components: valence and arousal. Valence refers to the level of behavioral activation either away from or toward a stimulus whereas arousal indicates the intensity of the emotional activation (Brücke et al., 2007). In this study, 10 DBS patients with PD viewed pleasant (positive valence), unpleasant (negative valence), and neutral visual stimuli matched for arousal chosen from the International Affective Picture System (IAPS) (Lang et al., 1999) while on anti-Parkinsonian medication. Compared to neutral stimuli, valenced stimuli resulted in larger alpha band (8–12 Hz) ERDs in the STN from 1 to 2 s after stimulus presentation. There were no significant differences between pleasant and unpleasant stimuli. In a follow up study with 9 PD DBS patients, the experiment was repeated for stimuli differing in both valence and arousal to show that individual alpha (7–13 Hz) ERDs are correlated with subjective valence ratings, but not arousal ratings (Brücke et al., 2007).

To separate STN emotional processing from the subsequent motor response that would occur in natural situations, a more recent study extended the above paradigm in several important ways (Buot et al., 2013). 16 PD patients viewed similarly valenced IAPS stimuli after STN DBS while both on and off anti-Parkinsonian medication. In addition to a passive-viewing condition, subjects responded with a button press to pleasant or unpleasant stimuli, thus representing a motor response to a valence-loaded stimulus. Larger ERPs were seen with emotional stimuli than neutral stimuli, regardless of their relevance for the motor portion of the task. That is, this effect was also observed during passive viewing. Interestingly, without medication, ERP amplitude was impacted by unpleasant stimuli but not by pleasant stimuli. This may suggest that encoding of pleasant stimuli partly requires an intact dopamine system (Huebl et al., 2014). Lastly, the authors examined the relationship between anatomical electrode location and ERP amplitude, showing a graded response that corroborates the tripartite notion of a distinct zone in the STN with a concentrated role in limbic processing (Alkemade et al., 2015).

These studies provided direct electrophysiological evidence for emotional processing within the STN. Next, Sieger and colleagues used single-neuron recordings in a study of 13 PD DBS patients (Sieger et al., 2015). In contrast to prior work, they discovered both valence and arousal-specific neurons in the STN. Surprisingly, the emotion-specific neurons identified in this study belonged to the dorsal sensorimotor region of the STN. This result thus challenges the tripartite notion of the STN and supports the possibility for meaningful measurements of non-motor function in what is traditionally considered motor territory of the basal ganglia (Rossi et al., 2015).

A limited number of studies have examined electrophysiological activity during auditory, as opposed to visual, emotional processing. A recent experiment presented auditory sentences to 15 PD patients a few days after their DBS procedures and analyzed STN LFP signals using ERP techniques (Péron et al., 2017). A spectral analysis was not included. As expected, sentences with positive (happy) or negative (angry) prosodies led to greater activity when compared to sentences with neutral prosody. In agreement with research suggesting that auditory decoding is largely a right-hemispheric process, this effect held true only for the right STN. Additionally, in a single unit study with 17 PD DBS patients, larger alpha (8–12 Hz) ERDs were seen in response to auditory stimuli with a positive or negative prosody, and in particular within the right ventromedial STN (Eitan et al., 2013).

Overall, the aforementioned studies signify the importance of the alpha frequency band for emotional processing in the STN. In fact, electrophysiological information within this band may have important clinical associations. A recent study by Huebl and colleagues investigated the correlation between alpha band ERD changes in response to valenced visual stimuli and depression scores as defined by the Beck Depression Inventory (BDI) in 12 PD DBS patients (Leentjens et al., 2000; Huebl et al., 2011). They defined an index of alpha ERD as the alpha ERD for pleasant stimuli minus the alpha ERD for unpleasant stimuli, and showed that it correlates with BDI 3 months following DBS surgery. In other words, reduced alpha ERDs may be used to predict patients more likely to experience depressive symptoms from DBS. Remarkably, this metric alone explained almost 50% of the variance of depressive symptoms. However, it should be mentioned that in this fairly small sample size, no patient developed severe depression after 3 months of chronic STN DBS. Nevertheless, such studies demonstrate the potential for clinical translation of electrophysiological data and provide further support for the meaningful role of the basal ganglia in non-motor processing.

## Response inhibition, conflict, and decision making

Within the motor domain, the brain must orchestrate prudent action selection in the face of numerous candidate actions to select among. Desirable and meaningful action selection hinges on intact response inhibition, a component process that the STN is perhaps best known for (Zavala et al., 2015). In this role, the STN directly and indirectly dictates whether a response to some stimulus is executed (Frank, 2006). To assess this decision-making process, studies typically employ behavioral tasks that present low-conflict and high-conflict trials in which decisions are relatively easy (i.e., requiring less inhibition) or hard (i.e., requiring more inhibition until a decision is made), respectively. The fundamental processes captured by such tasks—to execute appropriate decision-making—can be applied to both motor and non-motor systems (Aron et al., 2016). For instance, non-motor implications for an impaired generalized response inhibition may explain neuropsychiatric disorders such as impulsivity and may partly account for non-motor changes that occur after DBS (Weintraub and Zaghloul, 2013; Voon et al., 2017).

Together with the pre-supplementary motor area (pre-SMA) and prefrontal cortex (PFC), the STN plays a major role in behavioral control by acting as the breaking mechanism for the cortico-striatal driver network (Figure 1; Inase et al., 1999; Frank, 2006; Herz et al., 2014). This widely used analogy surpasses basic action control and can be generalized to include non-motor function as well. It has been previously established that medial PFC (mPFC) EEG theta power modulates with conflict and error (Cavanagh et al., 2009, 2010; Cohen and Cavanagh, 2011), and recent studies have extended these observations to LFP measures in the STN of PD DBS patients (Cavanagh et al., 2011; Alegre et al., 2013; Zavala et al., 2014). In one study, STN LFP signals were acquired intraoperatively during a low- and high-conflict image selection task (Cavanagh et al., 2011). During image selection tasks, subjects are conditioned to select one of two images on each trial to maximize probability of correct selection. Trials with a single image leading to a substantially larger probability of being the correct selection are deemed low-conflict trials, and those with two images of more similar probability are considered high-conflict trials. The STN showed heightened low-frequency theta (2.5–4 Hz) power following high-conflict cues, which has been replicated numerous times in studies using highly-conflicting stimuli (Brittain et al., 2012; Zavala et al., 2013, 2014). These findings are especially enlightening when considering the previously established connection between the mPFC and STN (Nambu et al., 2002), and the positive correlation between mPFC EEG theta power and slow response time during moments of high-conflict (Cavanagh et al., 2011).

A more precise understanding of the mPFC-STN link is afforded with invasive studies that record from both brain regions simultaneously. Zavala and colleagues examined STN LFP and mPFC EEG together in 13 PD patients with bilateral DBS implants (Zavala et al., 2014). Participants viewed moving dots on a computer screen and decided whether the dots were moving coherently toward the left or right. During high-conflict trials in which a minority subset of dots moved opposite to all other dots, the STN showed increased delta/theta activity (2–8 Hz). Notably, during high-conflict, the STN and mPFC were coherent within this delta/theta frequency band. Granger analysis showed a causal influence of the mPFC on the STN (Granger, 1969). These electrophysiological markers of inhibition vanished when participants provided their response by pressing the appropriate key on a keyboard. Taken together with single unit experiments, these studies provide electrophysiological biomarkers of the basal ganglia braking system involving the STN and mPFC during response inhibition (Frank, 2006; Zaghloul et al., 2012; Bastin et al., 2014). These results also align well with studies concluding a theta band communication mechanism between the mPFC and other cortical areas during conflict, such as the anterior cingulate cortex (Schroeder et al., 2002; Wang et al., 2005).

One of the most complex forms of high-conflict human decision-making is morality judgment, an evolutionarily recent ability linked primarily to social cognition. The concepts of morality and conflict are closely related, and since the deep brain participates in conflict evaluation through cortico-basal-thalamo-cortical circuits (Temel et al., 2005), it follows that morality judgment may rely on the basal ganglia. In a study of 16 PD DBS patients, LFP signals were recorded from the STN during morality evaluation using conflicting, non-conflicting, or neutral condition sentences (Fumagalli et al., 2011). The sentence content in this task exceeded that of basic emotional valence or prosody assessments used in studying emotional processing (see above). For instance, one sentence used in the task was “Some crimes must be punished by the death sentence.” Patients responded by pressing a button indicating if they agreed or disagreed with the sentence presented on each trial. These movements led to a decrease in beta (14–30 Hz) power, consistent with many motor studies of the STN (Marceglia et al., 2014). Interestingly, low-frequency (5–13 Hz) power increased during decision-making, more so during the conflict condition. Morality judgment certainly contains an emotional component (Greene et al., 2004; Fumagalli et al., 2010), but these results do not mimic those found in basal ganglia emotional evaluation (Kühn et al., 2005a; Brücke et al., 2007) because the conflicting and non-conflicting conditions in this study were balanced for emotional content. Rather, these results uniquely contribute to the body of evidence pointing to the basal ganglia's role in conflict processing through low-frequency oscillations.

Conflict and decision-making may also be assessed using the Stroop task. On each trial in this task, subjects view a colored word and indicate whether the word matches the color of the word (e.g., “red” in red font) or not (e.g., “blue” in red font). In one Stroop study, the STN beta band (15–35 Hz) modulated depending on correct or incorrect responding in 12 PD DBS participants (Brittain et al., 2012). Specifically, for high-conflict trials with word-color mismatch, beta power significantly increased prior to response for correct trials. In contrast, for incorrect trials, beta power only increased after responses occurred. Whereas the aforementioned studies implicate lower frequency signals in mediating a braking signal within the STN, these results point to a potential role for beta oscillations as well. While the beta frequency band is often associated with movement, cortical and basal ganglia beta changes have also been previously seen in the absence of movement during various behavioral tasks across different patient populations (Kühn et al., 2004; Swann et al., 2009; Ray et al., 2012; Bastin et al., 2014). Of note, increased theta during high-conflict trials was also seen in this study.

In addition to theta, alpha, and beta modulations, prior work furthermore suggests an important role for a gamma frequency (35–75 Hz) band during response inhibition. During a stop signal task in 10 PD DBS patients, Alegre and colleagues found that STN gamma decreased with successful inhibition (Alegre et al., 2013). Most interestingly, this effect was not seen in participants with clinically diagnosed impulse control disorder (ICD), a condition linked to impulsive responding from diminished inhibition (Rossi et al., 2015). While there were only 4 patients with ICDs in this cohort, and despite the complex relationship between response inhibition and multiple different frequency bands, these results also illustrate the potential for a clinical-translational impact of electrophysiological markers in the basal ganglia.

More recently, researchers recorded both intraoperative LFP and single unit spiking activity simultaneously from the STN of 15 PD patients while they performed a flanker task (Zavala et al., 2017). The flanker task asks subjects to indicate the direction of a central arrow which is flanked on either side by multiple other arrows. On high-conflict trials, the central arrow points opposite to all other arrows. Signals associated with stimulus processing were isolated from those related to the motor joystick response in this task. Theta (2–8 Hz) power significantly increased following high-conflict trials. Interestingly, individual neurons with task-related increased firing rates exhibited spike locking to beta (15–30 Hz) activity during high-conflict trials. These results may suggest that not only does beta power decrease to offer cells a chance to increase firing rates for subsequent movement (Courtemanche et al., 2003; Kühn et al., 2005b), but that beta-band spike locking may represent a purposeful processing delay for high-conflict evidence accumulation.

We point out that these numerous decision-making studies of the basal ganglia include a motor phase in which participants respond to a presented stimulus. For this reason, important limitations arise. For instance, to what degree these phenomena occur in the complete absence of movement remains understudied. However, studies take great measures to differentiate motor and non-motor components of each task and these signals are then analyzed separately. Despite this limitation, it is likely that response inhibition processing is shared among motor and non-motor processes alike (Bar-Gad et al., 2003; Weintraub and Zaghloul, 2013; Calabresi et al., 2014). Furthermore, it is important to note that investigations of response inhibition and high-conflict decision-making presented here are typically limited to studies of immediate actions and depend on the temporal sequence of the paradigm employed. For instance, a study of single units in humans showed that nucleus accumbens activity predicts future financial decisions that occur on average just 2 s later (Patel et al., 2012), however other studies use paradigms in which total trial duration may be <2 s (Rossi et al., 2017). From study to study, results cannot generally be applied to decisions occurring at different time scales (Zavala et al., 2015). Future studies that engage associative or limbic function without any anticipated near-future action could address these shortcomings.

## Error-detection and surprise

Acting as a guide for decision-making, error-detection is integral for successful goal-directed or Pavlovian-based learning. Error-detection refers to a surprising difference between expected and observed outcomes. In addition to several studies exploring error-detection at higher cortical areas (Schultz and Dickinson, 2000; Ridderinkhof et al., 2004; Oya et al., 2005; Asaad and Eskandar, 2011), a basal ganglia role for these processes is evident from many non-electrophysiological studies and several electrophysiological studies (García-García et al., 2017). Primate studies demonstrate that dopamine and striatum neurons code errors in predicted rewards (Schultz and Dickinson, 2000; Asaad and Eskandar, 2011). A DBS intraoperative study with OCD patients found STN single units with activity that modulate during error-monitoring of a stop-signal task (Bastin et al., 2014). Using a simple flanker task, a study with 9 dystonia patients found electrophysiological signatures of erroneous performance within the GPi (Herrojo Ruiz et al., 2014). Incorrect trials were associated with significantly larger GPi theta frequency (4–8 Hz) deviations, which were also seen at the posterior medial frontal cortex (pMFC) as measured by EEG. Also using a flanker task, Siegert and colleagues found positive STN LFP deflections between 260 and 450 ms after incorrect responding (Siegert et al., 2014). In a separate behavioral task of low and high conflict trials, STN low frequency oscillations (2.5–5 Hz) exhibited more phase consistency during the well-known phenomenon of post-error slowing, when reaction times increase following errors (Cavanagh et al., 2014).

Theta activity is certainly not limited to the basal ganglia, and neither is error processing. Zavala and colleagues focused their attention to the mPFC, a region known to be involved in error monitoring, and simultaneously examined brain signals from the STN during a flanker task (Zavala et al., 2016). Temporally aligned EEG signals from the mPFC and LFP signals from STN revealed that high-conflict trials led to a theta-delta band (2–8 Hz) coherence between the two structures, consistent with prior studies using high-conflict tasks (Zavala et al., 2014). Interestingly in this study, erroneous trials did not lead to theta power changes, but rather, higher phase coherence. Given the differing results seen from study to study, further work that specifically compares electrophysiological activity across trials with accurate and inaccurate responses are needed. Nonetheless, a theta frequency band generally appears to be involved in error detection.

While the phenomenon of surprise may be conceptually similar to error-detection, it is not necessarily associated with an expected event. Surprising stimuli or events may impact any ongoing cognitive and motor processing. At a neural level, the impact of surprise on the motor system is best explained through the fronto-basal ganglia network (Wessel et al., 2016). Since perturbations resulting from surprise are seen in both the motor domain (Wessel and Aron, 2013) and cognitive domain (such as diminished working memory encoding; Chiu and Egner, 2015), a recent study recorded basal ganglia LFPs during surprising events (Wessel et al., 2016). Namely, in response to unexpected tones played just before the retrieval phase of a verbal working memory task, the researchers found increased activity of the STN in PD patients across several frequency bands. However, given the nature of the task used in this study, it is difficult to determine whether the findings represent neural markers for a motor-specific, cognitive-specific, or global response to surprise-induced recruitment of the STN. Further studies should replicate these results across an array of non-motor processes.

## Reward processing

Reward processing encompasses the cognitive resources necessary for valuations of stimulus valence (attractiveness or aversiveness) in everyday life, including expectation, selection, and outcome (Rangel et al., 2008). From an electrophysiology standpoint, reward processing is one of the earliest studied non-motor functions of the basal ganglia, particularly within primates. Over four decades ago, researchers first noted neurons with activity specific to consummatory or reward-receipt stages of reward processing in primate striatum (Travis et al., 1968; Soltysik, 1975). This paved the way for one of the most comprehensive early studies of basal ganglia reward-processing, in which researchers examined single unit recordings in two macaques as they engaged in visuomotor tasks leading to liquid reward (Hikosaka et al., 1989). Caudate neurons responded to cue expectation, cue delay, expectation of a target as indicated by a cue, expectation of reward regardless of the task needed to obtain reward, motor actions necessary to receive reward, including motor preparation, as well as to reward itself. These findings laid the groundwork for subsequent studies further investigating specific components of this reward-processing spectrum. For example, similar primate studies showed striatal single unit activity associated with the expectation of a reward and reward receipt (Bowman et al., 1996; Hollerman et al., 1998; Lauwereyns et al., 2002; Cromwell and Schultz, 2003; Darbaky et al., 2005; Espinosa-Parrilla et al., 2015), regardless of whether reward was obtained via movement of the hand or by withholding movement, such as by maintaining fixation on a target (Apicella et al., 1991, 1992; Matsumura et al., 1992; Schultz et al., 1992). Notably, neurons in basal ganglia regions traditionally considered to be purely motor nuclei, including the dorsal STN, have been shown to modulate during anticipatory reward stages (Darbaky et al., 2005; Espinosa-Parrilla et al., 2013). Certain striatal neurons may also be conditioned to respond exclusively to cues that are not rewarded (Kawagoe et al., 1998). Neurons in primate anterior striatum differentially respond to rewards based on their reward magnitudes (Hollerman et al., 1998; Hassani et al., 2001; Cromwell and Schultz, 2003). Overall, reward-related neurons in the primate basal ganglia respond to an impressive variety of reward processing stages and contexts.

Numerous modifications to reward tasks such as the delayed response or Go/No-Go behavioral paradigms have been employed to rigorously test reward-processing in primates and control for confounding factors. For instance, one study showed that although striatal neurons code for cues that signify future rewards following consistent temporal delays, this effect wanes for cues that instead lead to reward receipt with a random delay of time (Shidara et al., 1998). Aside from visual cues, one study demonstrated that tonically active striatal neurons in two macaques can be conditioned to respond to an auditory cue associated with a liquid reward (Aosaki et al., 1994). These neurons were also capable of responding to multiple different rewards. To further demonstrate that neurons responding to reward delivery do not specifically depend on movement, Darbaky et al. varied the reward timing and showed that 13 of 15 reward-responsive neurons accordingly displaced their response activity (Darbaky et al., 2005). A more recent primate study of reward processing characterized STN neurons during reward receipt both independent of, and dependent on, motivational-based choice selection of juice or water reward (Espinosa-Parrilla et al., 2015). STN neuron activity reflected whether or not the animals had received their preferred reward.

In relationship to other brain regions, to what extent does the basal ganglia encode reward-related information? Since the orbitofrontal cortex (OFC) is networked to the basal ganglia for motivational coding (Selemon and Goldman-Rakic, 1988; Haber et al., 1990; Eblen and Graybiel, 1995; Li et al., 2016), Schultz et al. used several behavioral paradigms during monkey single unit recordings to compare striatal and OFC neurons during reward processing (Schultz et al., 2000). They found that while OFC neurons respond to rewarding cues, reward expectation just prior to reward receipt, and reward receipt, striatal neurons have more extensive action-related reward responses; that is, striatal neurons additionally respond during reward-related motor preparation and action. In both regions, neurons discriminated between specific types of liquid or food rewards. Neurons in primate dorsolateral prefrontal cortex also modulate with reward expectancy (Watanabe, 1996). In addition to the ventral striatum and pallidum, which encode expected reward in primates (Tachibana and Hikosaka, 2012), another region strongly implicated in limbic reward processing is the lateral habenula (LHb) with afferent input from the GPi (Matsumoto and Hikosaka, 2007; Hong and Hikosaka, 2013). It is not surprising then, that the GPi in monkeys shows activity modulated by reward expectation and no-reward expectation (Hong and Hikosaka, 2008), particularly throughout the dorsal and ventral border. Furthermore, these neurons have a unique firing pattern with respect to spike rate and spike duration that differs from motor GPi units. Reward prediction neurons can also be found in primate globus pallidus externus (GPe) (Arkadir et al., 2004). Finally, human electrophysiological studies have also advanced the previously well-recognized role of the nucleus accumbens as a reward center in the brain. Oscillatory activity recorded directly from the nucleus accumbens in 5 patients undergoing DBS for major depression showed that bursts of gamma activity (40–80 Hz) tended to occur during peaks of coexisting alpha activity (8–12 Hz) (Cohen et al., 2009). Moreover, rewards given during a behavioral task adjusted the timing of this relationship. Further studies are needed to confirm if this cross-frequency coupling represents the electrophysiologic signature of the hypothesized mechanism of the nucleus accumbens as the link between the limbic system and the basal ganglia (Day and Carelli, 2007).

It is well-known that that dopamine neurons encode many aspects of reward processing (see Schultz, 1998 for a review) and that dopamine status impacts reward processing ability. Similar to striatal neurons, primate dopamine neurons respond to reward expectation stimuli, reward receipt, and reward prediction error (Mirenowicz and Schultz, 1996; Schultz and Dickinson, 2000), separate from movements (Romo and Schultz, 1990). Given a stimulus indicating a probability of reward delivery, uncertainty (i.e., low probability) corresponds to increased dopamine and the phasic activity of dopamine corresponds to the reward prediction error (Fiorillo et al., 2003). ICDs are also closely related to impaired reward processing in PD patients, and ICDs can result from high levels of dopamine intake in PD, or occasionally after DBS (Rossi et al., 2015). In one study, PD patients with pathological gambling who used a risky strategy during a gambling task showed that while all PD patients exhibited STN low frequency power (2–12 Hz) increases during economics decisions, these increases were seen more during high-risk than low-risk decisions (Rosa et al., 2013). A recent study of one patient with OCD demonstrated increases in delta LFP power in the nucleus accumbens during reward anticipation (Wu et al., 2017). In the ON-dopamine state, but not in the OFF-dopamine state, 9 PD DBS patients with ICDs and without dopamine-induced dyskinesias showed elevated STN theta (4–7.5 Hz) activity coherent to EEG theta in the premotor and frontal cortex (Rodriguez-Oroz et al., 2010). In contrast, PD patients with dyskinesias showed coherent alpha frequency (7.5–10 Hz) activity between the STN and primary and supplementary motor cortices. It should be noted that in this relatively small sample, patients retrospectively self-completed ICD assessments and had few neuropsychiatric control variables. Accurate diagnosis of ICDs remains problematic, especially since individuals with ICDs tend to underreport their impulsive natures (Papay et al., 2011), and impulsivities may present in many different manners. Nonetheless, this work reaffirms that basal ganglia LFP signals could have clinically meaningful non-motor associations, especially since reward correlates, as reviewed here, can be measured at multiple nodes of the basal ganglia reward system.

The majority of the above studies considered action and inaction conditions for reward receipt, but outcomes were generally either neutrally valenced (i.e., not rewarded) or positively valenced (e.g., liquid reward). Overall, dopamine and pallidal neurons in primates preferentially respond to appetitive stimuli as opposed to aversive stimuli (Mirenowicz and Schultz, 1996; Joshua et al., 2009). In humans, negative valence (i.e., threat of loss or punishment) and single unit responses to reward were first investigated in a recent study of the STN and GPi during a modified Go/No-Go task (Rossi et al., 2017). While both structures showed neurons responsive to reward, this large study of 53 PD DBS patients moreover demonstrated that regardless of the valence-related stimulus—whether being a reward receipt or successful avoidance of loss—the STN modulated more than the GPi. Most of the neurons identified, which were distributed throughout both structures in a non-tripartite fashion, were exclusive for reward anticipation, reward receipt, threat of loss, or successful avoidance of loss. Another recent study in 8 patients—four with Tourette syndrome, one with generalized dystonia, two with cervical dystonia, and one with dystonic tremor—supports the role of the GPi in reward processing (Münte et al., 2017). Specifically, LFP oscillatory changes were seen in the high beta range (20–30 Hz) at approximately 500 ms after rewards, but not losses. Future studies should extend these findings to the STN for comparison, and examine LFP changes during reward expectation and during non-movement conditions.

Given that reward-related cognitive processing ultimately inform learning processes for improved decision-making, numerous studies have integrated learning into reward-processing tasks (Romo and Schultz, 1990; Ljungberg et al., 1992; Aosaki et al., 1994; Tremblay et al., 1998; Tremblay and Schultz, 2000). In these paradigms, basal ganglia neurons are recorded during conditioning phases in which primates learn to pair new cues and subsequent movements with a reward. During this learning phase, reward-expecting striatal neurons activate regardless of the presence or absence of actual reward receipt, but they rapidly begin to activate only for rewarded movements (Tremblay et al., 1998). Similar to reward anticipation, activity of certain neurons also either decrease or increase specifically during the learning phase. Neurons throughout the GPi—not only within specific anatomic subregions—fire differentially when primates learn new visuomotor associations and when they exploit that new information (Sheth et al., 2011; Gale et al., 2014). During exploitation, GPi neurons exhibit higher firing rates to suppress competing actions (see Figure 1). In dopamine neurons, depression typically occurs at the expected time of reward receipt when predictable rewards unexpectedly do not occur (Ljungberg et al., 1991; Schultz et al., 1993). It has been shown in multiple studies that dopamine neurons respond to reward receipt primarily in the context of unpredictability, likely reflecting a mechanism of learning, whereas dopamine neuron activations occur less after conditioning has been established (Romo and Schultz, 1990; Ljungberg et al., 1992; Mirenowicz and Schultz, 1994).

Learning processes likely utilize the full extent of the basal ganglia functional substructures. For instance, the ventral striatum is linked with incentive motivational behaviors for informative goal-directed behavior executed in the dorsal striatum–coordinated activity of these regions enhance learning performance (Katnani et al., 2016). Beyond the deep brain, the basal ganglia is particularly well-suited to be involved in learning through its interconnections with numerous higher cortical areas. However, the exact manner in which the basal ganglia interacts with these higher cortical areas, and in what temporal order, remains under investigation. In one study, reward-related firing of anterior cingulate neurons in humans predicted motor responses before they occurred (Williams et al., 2004). When monkeys learn to pair a saccade direction with reward-receipt, a study of 432 prefrontal cortex (PFC) and 279 caudate nucleus neurons showed that caudate nucleus activity coded for reward-associated saccade direction sooner than the PFC (Pasupathy and Miller, 2005). A similar monkey sensorimotor learning task involved reaching as opposed to saccades, and demonstrated that while dorsal premotor cortex and basal ganglia neurons modulate with learning novel cue-reward pairings, their timing is not different (Brasted and Wise, 2004). Either the basal ganglia and cortical learning areas are involved in separate learning processes altogether, or they represent a shared learning network with an ordinal layout that is not yet fully resolved.

## Language

From a historical perspective, the neural bases of language function have been mostly linked to the neocortex (Broca, 1861; Wernicke, 1874). It was not until the twentieth century when evidence of language dysfunction was reported in patients with ischemic and hemorrhagic basal ganglia lesions (Hier et al., 1977; Damasio et al., 1982; Wallesch, 1985). Animal studies further corroborated the relationship between the basal ganglia and language cognition for syntax, learned behavior, and sequencing (Aldridge et al., 1993; Graybiel, 1995; Berns and Sejnowski, 1998), but these inferences could not be directly extrapolated to humans (Lieberman, 2009).

Electrophysiological investigations of basal ganglia language function began in the 1980s. For instance, morphosemantic (meaning) mismatches elicit negative-going N400 waves in the sensorimotor cortex (Kutas and Hillyard, 1980; Holcomb and Neville, 1990), and syntactic inconsistencies consistently elicit a P600 wave (Osterhout and Holcomb, 1992) preceded by early left anterior negativity (ELAN) ERPs in the parietal and frontal cortical regions, respectively (Hahne and Friederici, 2001). By studying patients with basal ganglia lesions and neurodegenerative diseases like PD during language tasks, and by comparing their EEG correlates with healthy patients, scientists had an opportunity to potentially infer linguistic roles of the basal ganglia. In a series of experiments, patients with basal ganglia lesions and PD completed syntactic (ELAN/P600) and semantic (N400) violation tasks. In comparison to healthy controls, these patients elicited delayed N400 ERPs (Kotz et al., 2003; Angwin et al., 2017), as well as modulations of the P600 wave (Hahne and Friederici, 1999; Friederici et al., 2003; Kotz et al., 2003). However, the extent to which these phenomena result from basal ganglia substrate remains unclear. In a follow-up study combining EEG and DBS lead recordings, the STN and the GPi, unlike the ventral intermediate nucleus of the thalamus, did not show characteristic ERPs during analogous syntactic and semantic violations (Wahl et al., 2008). Nonetheless, the basal ganglia is likely involved in aspects of language processing, a possibility widely supported by extensive PD and PD-DBS longitudinal language assessments (Parsons et al., 2006; Wojtecki et al., 2006; Okun et al., 2009; Mikos et al., 2011; Yamanaka et al., 2012) and intraoperative electrophysiological data from single unit and DBS LFP recordings.

Among the initial direct electrophysiological evidence of STN modulation during language tasks came from the work of Watson and Montgomery, who performed intraoperative single unit recordings of PD DBS patients during a simple reading task. High levels of spiking in the STN were generated just before voice onset that correlated to the pronunciation of each syllable (Watson and Montgomery, 2006). The verbal fluency task is commonly used to assess both phonemic and semantic eloquence. This task has two distinct phases with specific timings. The phonemic phase asks individuals to generate as many words that begin with a given letter excluding proper nouns or variants of a previously mentioned word. On the other hand, the semantic phase asks participants to produce as many words as possible that correspond to a particular semantic classification (e.g., colors or countries). Using intraoperative STN LFP recordings during a semantic and phonemic verbal fluency task, Anzak and colleagues found significant increases in gamma (30–95 Hz) power as well as decreases in beta (13–30 Hz) power. Interestingly left-hemispheric gamma power correlated with correct responses when the patient transitioned or switched from one semantic classification to another in the semantic phase or from one letter to the next in the phonemic phase. These results suggest that the STN plays a role in switching during verbal fluency (Anzak et al., 2011). Most recently, Wojtecki and colleagues reported significant power increases in the alpha-theta range (6–12 Hz) of STN LFP activity during a verbal generation task. It was also observed that electrodes located closer to the ventromedial STN had the greatest power responses to the verbal generation task. Likewise, coherence analysis between frontotemporal surface EEG electrodes and STN LFPs showed significant power increases at 6–7 Hz (Wojtecki et al., 2017).

In order to properly distinguish the motor and silent internal components of speech production, one study combined an analogous speech task with a finger tapping motor task. Intraoperative STN LFP signals showed that tasks involving both language and motor activity had high beta (13–30 Hz) power modulation. Conversely, combination of overt and imaginary speech was associated with low beta levels, demonstrating modulation independency between speech and motor function (Hebb et al., 2012). Similarly, EEG and ECoG experiments have reported cortical beta oscillations associated with word production (Crone et al., 2001; Edwards et al., 2009). Furthermore, using surface EEG and STN-LFP, Hohlefeld et al. (2017) found that lexical accuracy correlates with cortico-subthalamic coherence in the beta (14–35 Hz) range (Hohlefeld et al., 2017). These results indicate existence of non-motor synchronization of the cortex and basal ganglia in language production. Future studies are still needed to assess whether electrophysiological markers of language in the basal ganglia provide clinically meaningful associations, for instance, to language perturbations commonly seen after DBS.

## Time processing

Several lines of evidence suggest that the basal ganglia play an important role in beat-based timing via a thalamo-cortical-striatal circuitry (Meck and Benson, 2002; Teki et al., 2011). For instance, PD and Huntington's disease patients have low performance in temporal reproduction tasks (Malapani et al., 1998; Paulsen et al., 2004),(Allman and Meck, 2011). Similar internal clock deficiencies are seen after induced damage of dopaminergic pathways (Buhusi and Meck, 2002; Chang et al., 2007). It is hypothesized that substantia nigra dopaminergic output modulates oscillatory activities within striatal medium spiny neurons (Buhusi and Meck, 2005). These dorsal striatal neurons may act as coincidence detectors of cortical oscillations to encode beat-based timing. In PD DBS postoperative studies, multiple studies, including a double-blind experiment, have shown significant improvements in duration estimates after STN DBS (Koch et al., 2004; Wojtecki et al., 2011).

Direct neuroelectrophysiological evidence of the role of the basal ganglia in time processing comes from the experiments of Bartolo and colleagues in non-human primates. In these experiments, monkeys were trained to perform a synchronization continuation task (SCT). This task consists of tapping a button three times in synchrony with a guidance isochronous metronome, followed by a continuation phase in which the monkey has to tap the buttons at the same time intervals but with no metronome or external cues. Putaminal single unit and LFP recordings showed beta (15–30 Hz) coherent activity among distant electrodes and increased beta modulation when the animal had to internally temporalize its rhythmic activity (post-synchronization phase). Likewise, gamma (30–70 Hz) frequency had a slightly increased burst modulation during the metronome guided (synchronization) period of the task (Bartolo et al., 2014).

Following these findings in putaminal LFP activity, the hypothesis that serial rhythmic striatal beta modulation might be involved in non-periodic movements was tested (Teki et al., 2011). In these subsequent experiments, striatal oscillatory activity was evaluated during a SCT task in combination with a serial reaction time task (RTT), which differed from the STC by having irregular tapping sequences. Transient beta modulation was observed throughout both regular (STC) and irregular (RTT) tasks. Remarkably, beta power considerably increased during the continuation phase of the SCT, as well as during the initial phase of the RTT task. These results suggest involvement of the basal ganglia in giving the initiating prompt of behaviors involving sequential timing (Bartolo and Merchant, 2015).

## Limitations

Important limitations across these electrophysiology studies must be noted. With few exceptions, this research in humans has relied on DBS patient populations to explore basal ganglia function. As a result, in many cases extrapolation to normal healthy populations is not easily justified (Marceglia et al., 2009). These studies primarily recruit from diseased populations undergoing surgical treatment. During awake- neurosurgery signal acquisitions, patients may be anxious or uncomfortable (Patel et al., 2013). With repeat studies using different diseased populations, basal ganglia LFP studies may become more generalizable. Currently, control populations are critically missing. In addition, the majority of these studies collect electrophysiological signals from a single anatomical region in the brain. For instance, a study with human PD patients undergoing STN DBS may collect electrophysiological data only from the dorsolateral STN. This presents as a main setback for exploring non-motor function, especially given the notion of a tripartite basal ganglia as discussed above. At the same time, accurately quantifying specific DBS lead locations after implantation remains difficult, particularly normalization processes for purposes of meaningful comparison across different patients.

It is also important to consider that the exact nature of LFPs remain unclear and that they can arise from many sources across many sites, even from locations where units are not firing (Herreras, 2016). For instance, LFP dynamics can be heavily influenced from afferent activity in the recorded area (Herreras, 2016). In addition, specific neuronal signal acquisition and interpretation are heavily dependent on recording strategies and analytic approaches. Each electrophysiological recording modality also has its own set of technical setbacks. For example, EEG recordings of patients with DBS implants are impacted by burr holes (Bénar and Gotman, 2002; Oostenveld and Oostendorp, 2002), and MEGs suffer from high-amplitude artifacts from percutaneous extension wires (Litvak et al., 2011). These methodological limitations will be increasingly overcome with advanced surgical techniques, electrophysiology equipment, and analysis tools.

## Conclusion

We have reviewed an extensive literature on non-motor electrophysiology of human and non-human primate basal ganglia, primarily from single-unit and LFP studies. Beyond neuroscience discovery, such neural correlates can be used to implement therapies such as brain computer interfaces. Microelectrode recordings of single-units have revealed fundamental basic neurophysiology of subcortical structures, but continued LFP characterization of non-motor processing remains critical for clinical translation. For instance, macroelectrodes implanted in the basal ganglia for movement and neuropsychiatric disorders reliably record LFPs—not single-units—and their electrophysiological capabilities are more resistant to the foreign body response (Vetter et al., 2004; Koivuniemi et al., 2011; Urdaneta et al., 2017; Wong et al., 2018).

**Figure 3:**
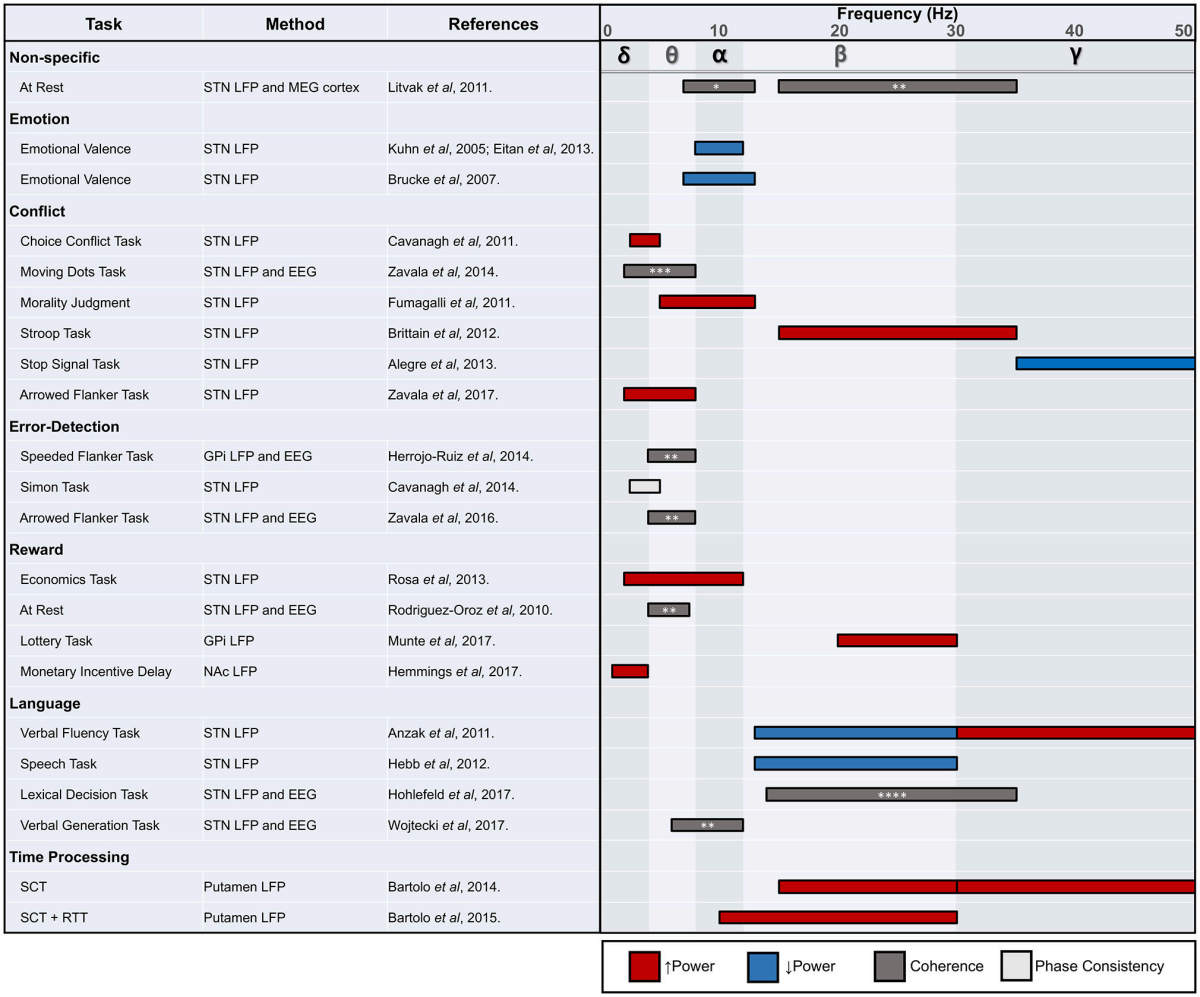
STN, subthalamic nucleus; LFP, local field potential; MEG, magnetoencephalography; EEG, electroencephalography; GPi, globus pallidus internus; NAc, nucleus accumbens; SCT, synchronization continuation task; RTT, reaction time task. ^*^ tempoparietal EEG, ^**^ frontal EEG, ^***^ causal influence on mPFC, ^****^ frontal-central-parietal EEG. Review of human non-motor electrophysiology studies of the basal ganglia. Approximate locations for commonly identified oscillatory frequency bands are included.

A summary of findings from human non-motor basal ganglia LFP studies is provided in Figure 3. While most human basal ganglia LFP studies to date have investigated response inhibition and conflict, more work is needed in other areas of non-motor function, particularly those with clinically relevant underlying deficits across different disease pathologies like reward processing dysfunction in PD or OCD. Furthermore, informative and underutilized methodological approaches, such as simultaneous invasive and non-invasive recordings or spike-field coherence, should be further applied to other non-motor domains.

While limited in number and variable in scope, the LFP studies reviewed here point to multiple frequencies of interest outside of the beta range, which has become a widely explored band for motor function. In general, as we have seen throughout this review, lower frequency activity may be most consistently attributed to non-motor processing (Litvak et al., 2011). The exceptions to this rule of thumb are not rare in number, however, and require careful consideration in future work. Beta modulation can also be seen in the absence of movement, and higher frequencies, including those in the gamma range, appear related to several non-motor domains. It would be premature to conclude that the bulk of non-motor processing in the basal ganglia occurs in frequencies below the beta range, although multiple studies now support this hypothesis. Nonetheless, there now exists unequivocal electrophysiological evidence that the basal ganglia are involved in non-motor function. The treatment of several psychiatric and movement disorders with DBS in particular has enabled the investigation of deep brain structures and their relationship to various behaviors. Although electrophysiological correlates of movement are well established, the precise role of the basal ganglia in non-motor function requires many more studies. Future work should focus on associations between electrophysiological markers and clinical outcomes, which has proven to be a fruitful line of study for motor symptoms in conditions such as PD. Moving forward, these translational studies have great potential to uncover actionable pathological signals that can be targeted for clinical care through neuromodulation.

## Author contributions

RE conceptualized the paper. RE wrote the first draft with the exception of sections 8 and 9. MU wrote the first draft of sections 8 and 9. MU developed the first drafts of the figures. MU and RE finalized figures. RE, MU, KF, MO, and AG provided critical input and edits.

### Conflict of interest statement

The authors declare that the research was conducted in the absence of any commercial or financial relationships that could be construed as a potential conflict of interest.
